# Liraglutide in mild to moderate Alzheimer’s disease: a phase 2b clinical trial

**DOI:** 10.1038/s41591-025-04106-7

**Published:** 2025-12-01

**Authors:** Paul Edison, Grazia Daniela Femminella, Craig Ritchie, Joseph Nowell, Clive Holmes, Zuzana Walker, Basil Ridha, Sanara Raza, Nicholas R. Livingston, Eleni Frangou, Sharon Love, Gareth Williams, Robert Lawrence, Brady Mcfarlane, Hilary Archer, Elizabeth Coulthard, Benjamin R. Underwood, Paul Koranteng, Salman Karim, Carol Bannister, Robert Perneczky, Aparna Prasanna, Kehinde Junaid, Bernadette McGuinness, Ramin Nilforooshan, Ajay Macharouthu, Andrew Donaldson, Simon Thacker, Gregor Russell, Naghma Malik, Vandana Mate, Lucy Knight, Sajeev Kshemendran, Christian Holscher, Anita Mansouri, Mae Chester-Jones, Jane Holmes, Trisha Tan, Steve Williams, Azhaar Ashraf, David J. Brooks, John Harrison, Rainer Hinz, George Tadros, Anthony Peter Passmore, Clive Ballard

**Affiliations:** 1 Faculty of Medicine, Imperial College London, London, UK; 2 School of Medicine, College of Biomedical and Life Sciences, Cardiff University, Cardiff, UK; 3 Scottish Brain Sciences, Edinburgh, UK; 4 Southern Health NHS Foundation Trust, Havant, UK; 5 University College London and Essex Partnership University NHS Foundation Trust, Runwell, UK; 6 Brighton and Sussex University Hospitals NHS Trust, Brighton, UK; 7 MRC Clinical Trials Unit at UCL, London, UK; 8 Kingʼs College London, London, UK; 9 SW London and St. George’s Mental Health NHS Trust, London, UK; 10 North Bristol NHS Trust, Bristol, UK; 11 Bristol Medical School, University of Bristol, Bristol, UK; 12 Department of Psychiatry, University of Cambridge, Cambridge, UK; 13 Northamptonshire Healthcare NHS Foundation Trust, Kettering, UK; 14 Lancashire Care NHS Foundation Trust, Preston, UK; 15 Black Country Partnership NHS Foundation Trust, West Bromwich, UK; 16 Nottinghamshire Healthcare NHS Foundation Trust, Nottingham, UK; 17 Centre for Public Health, Queen’s University Belfast, Belfast, UK; 18 Surrey and Borders Partnership NHS Foundation Trust, Leatherhead, UK; 19 NHS Ayrshire and Arran, Crosshouse, UK; 20 NHS Lanarkshire, Glasgow, UK; 21 Derbyshire Healthcare NHS Foundation Trust, Derby, UK; 22 Bradford District Care NHS Foundation Trust, Bradford, UK; 23 5 Boroughs Partnership NHS Foundation Trust, Warrington, UK; 24 Cornwall Partnership NHS Foundation Trust, Redruth, UK; 25 Somerset Partnership NHS Foundation Trust, Bridgwater, UK; 26 South Staffordshire and Shropshire Healthcare NHS Foundation Trust, Stafford, UK; 27 Henan University of Chinese Medicine, Zhengzhou, China; 28 University of Oxford, Oxford, UK; 29 Newcastle University, Newcastle upon Tyne, UK; 30 Aarhus University, Aarhus, Denmark; 31 Metis Cognition, Ltd., Kilmington, UK; 32 Alzheimer Center, Amsterdam UMC, Amsterdam, The Netherlands; 33 University of Manchester, Manchester, UK; 34 Aston Medical School, Aston University, Birmingham, UK; 35 University of Exeter, Exeter, UK

**Keywords:** Cell death in the nervous system, Alzheimer's disease

## Abstract

Liraglutide, a glucagon-like peptide 1 (GLP-1) agonist and antidiabetic drug, has shown neuroprotective effects in animal models. In this study, we aimed to evaluate the safety and efficacy of liraglutide in mild to moderate Alzheimer’s disease syndrome. ‘Evaluating liraglutide in Alzheimer’s disease’ (ELAD) is a multicenter, randomized, double-blind, placebo-controlled phase 2b trial in 204 participants with mild to moderate Alzheimer’s disease syndrome with no diabetes. Participants received daily injections of liraglutide or placebo for 52 weeks. They underwent fluorodeoxyglucose positron emission tomography, magnetic resonance imaging and detailed neuropsychometric evaluations. The primary outcome was a change in cerebral glucose metabolic rate. Secondary outcomes were safety and tolerability and cognitive changes. The primary outcome showed no significant differences in cerebral glucose metabolism (difference = −0.17; 95% confidence interval: −0.39 to 0.06; *P* = 0.14) between the two groups. The secondary outcome—score on the Alzheimer’s Disease Assessment Scale-Executive domain (ADAS-Exec)—performed better in liraglutide-treated patients compared to placebo (0.15; 95% confidence interval: 0.03−0.28; unadjusted *P* = 0.01). No significant differences were observed in Alzheimer’s Disease Cooperative Study-Activities of Daily Living (ADCS-ADL) (−0.58; 95% confidence interval: −3.13 to 1.97; unadjusted *P* = 0.65) or Clinical Dementia Rating-Sum of Boxes (CDR-SoB) (−0.06; 95% confidence interval: −0.57 to 0.44; unadjusted *P* = 0.81) scores. Liraglutide was generally safe and well tolerated in non-diabetic patients with Alzheimer’s disease. ClinicalTrials.gov identifier: NCT01843075.

## Abstract (web summary)

Results from the phase ELAD 2 trial reveal that liraglutide is safe and well tolerated in people with mild to moderate Alzheimer’s disease but does not significantly slow brain metabolism decline.

## Main

Alzheimer’s disease is characterized by multiple pathologies, including β-amyloid deposition, tau aggregation, neuroinflammation/glial activation and synaptic dysfunction, which contribute to progressive neurodegeneration. For an effective treatment, a multitargeted approach influencing these different pathologies may be required. The GLP-1 receptor agonist liraglutide has shown compelling preclinical evidence of influencing multiple targets in transgenic mouse models. Liraglutide has 97% homology to human GLP-1 and is currently approved worlwide for treating type 2 diabetes and obesity^1^.

In transgenic mouse models of Alzheimer’s disease, liraglutide improves memory, prevents synaptic loss, reduces β-amyloid and tau aggregation, reduces neuroinflammation and oxidative stress, restores protein kinase A and phosphoinositide 3‑kinase/protein kinase B signaling and improves insulin signaling^2–4^. Liraglutide increases stem cell proliferation and differentiation into neurons^5^, enhancing neurogenesis in the dentate gyrus^6^ and preventing memory decline^7^.

In one study evaluating dulaglutide, a GLP-1 analog, patients with diabetes showed delayed cognitive impairment (*n* = 9,901) compared to placebo^8^. Another pooled post hoc analysis of three large cardiovascular outcome trials revealed that liraglutide and semaglutide significantly reduced the incidence of dementia^9^. Liraglutide significantly prevented the decline of brain glucose metabolism in a pilot study involving 38 patients with Alzheimer’s disease^10^. Another small study involving at-risk participants demonstrated that liraglutide improved intrinsic connectivity within the default mode network^11^. A trial of the GLP-1 analog exenatide in Alzheimer’s disease found a reduction of β-amyloid 42 in plasma neuronally derived extracellular vesicles^12^. Systematic reviews and the Delphi consensus in 2012 and 2020 highlighted GLP-1 agonists as the most promising class of compounds for repurposing as a potential therapy for Alzheimer’s disease^13^. Another recent study evaluating liraglutide in Parkinson’s disease improved non-motor symptom scores, Movement Disorders Society Unified Parkinson’s Disease Rating Scale (MDS-UPDRS) part II scale and the 39-item Parkinsonʼs Disease Questionnaire (PDQ-39)^14^. Exenatide treatment improved MDS-UPDRS scores in participants with Parkinson’s disease compared to controls^15,16^. Motor and cognitive improvements were maintained 12 months after exenatide treatment cessation^17^. Analysis from neuronal-derived exosomes shows that exenatide engaged insulin, Akt and mTOR signaling pathways^18^. A phase 2 trial showed that 12 months of lixisenatide treatment improved MDS-UPDRS part III scores relative to placebo^19^.

In the ELAD study, we sought to evaluate the influence of liraglutide on the change in cerebral glucose metabolic rate (rCMRglc), cognition and magnetic resonance imaging (MRI) volume from baseline to week 52 in patients with mild to moderate Alzheimer’s disease syndrome.

## Results

### Patient disposition

**Fig. 1:**
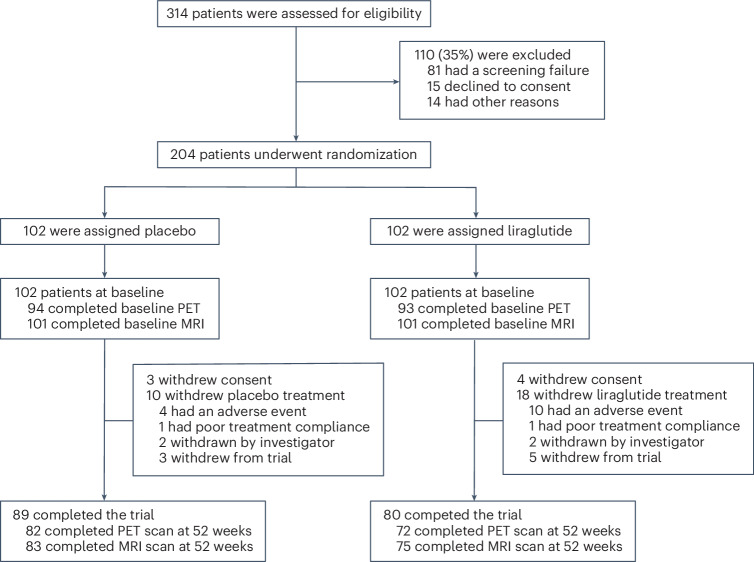
Consort diagram. Study procedures: patient enrollment and randomization. PET, positron emission tomography.

Of the 204 participants randomized, 102 were assigned to receive liraglutide and 102 to receive placebo. In total, 169 participants completed the study. Analyzable scans at week 52 for the primary outcome were 72 participants (70.6%) in the treatment arm and 82 participants (80.4%) in the placebo arm. The most common reasons for participants not completing the trial included withdrawal of consent, clinical decision, poor compliance, adverse events and participants unable to tolerate scans (Fig. 1). The participants in the treatment and placebo groups were generally similar in terms of baseline demographics and clinical characteristics (Table 1). Female sex (38% versus 41%), age (72.5 years versus 70.6 years) and education (12.9 years versus 13.1 years) were balanced in the placebo and treatment groups, respectively. Four participants identified as non-White, and the remainder identified as White. A similar number of patients with Mini-Mental State Examination (MMSE) scores ≤18 (*n* = 13 for placebo and *n* = 10 for treated) and MMSE scores >18 (*n* = 89 for placebo and *n* = 92 for treated) were included (Supplementary Table 1). Moreover, a similar number of participants with CDR scores of 0.5 (*n* = 60 for placebo and *n* = 61 for treated), 1 (*n* = 38 for placebo and *n* = 40 for treated) and 2 (*n* = 2 for placebo and *n* = 1 for treated) were included (Supplementary Table 2).

**Table 1:** Baseline characteristics^a^

IQR, interquartile range	Placebo (*n* = 102)	Treatment (*n* = 102)	Total (*n* = 204)	*P* value (placebo versus treatment)
Female sex (no. (%))	39 (38)	42 (41)	81 (40)	0.6680
Age (years)^b^	72.5 ± 7.0	70.6 ± 8.4	71.5 ± 7.8	0.0808
Education (years)	12.9 ± 3.0	13.1 ± 4.2	13.0 ± 3.7	0.6960
MMSE score^b,c^	23.4 ± 3.6	23.7 ± 3.5	23.5 ± 3.6	0.5469
ADAS-Cog 13 score^d^	31.9 ± 9.3	31.6 ± 10.3	31.8 ± 9.8	0.8274
CDR-SoB score^e^	3.6 ± 1.8	3.7 ± 1.9	3.7 ± 1.9	0.7000
ADCS-ADL score^f^	66.1 ± 9.7	66.5 ± 9.5	66.3 ± 9.5	0.7664
Neuropsychiatric Inventory (NPI)	7.5 ± 9.0	10.2 ± 12.5	8.9 ± 10.9	0.0783
Geriatric Depression Scale (GDS)	5.5 ± 4.4	5.4 ± 4.0	5.4 ± 4.2	0.8653
Controlled Oral Word Association Test (COWAT)	11.5 ± 4.9	9.8 ± 4.2	10.7 ± 4.6	0.0084
Category Fluency Test (CFT)	10.5 ± 4.8	10.3 ± 5.5	10.4 ± 5.1	0.7823
Trail Making Test Part A (TMT-A) score	71.2 ± 52.3	78.2 ± 58.0	74.8 ± 55.3	0.3664
Trail Making Test Part B (TMT-B) score	148.8 ± 78.4	149.0 ± 80.6	148.9 ± 79.3	0.9857
Wechsler Digit Span Forward (WDS-F) score	8.8 ± 2.2	8.0 ± 2.4	8.4 ± 2.3	0.0139
Wechsler Digit Span Backward (WDS-B) score	5.3 ± 2.3	5.0 ± 2.2	5.1 ± 2.3	0.3423
Insulin (median (IQR))	6.2 (4.1−8.7)	6.9 (4.4−9.1)	6.5 (4.2−9.1)	0.3400
Lipase (U l^−1^) (median (IQR))	40.5 (34.0−53.0)	46.0 (33.0−68.5)	43.0 (34.0−60.0)	0.002
Amylase (U l^−1^)	80.1 ± 50.3	80.7 ± 58.3	80.4 ± 54.3	0.9374

^a^The data in the table are based on the baseline visit data unless the variable was part of the exclusion/inclusion criteria or recorded only at screening.

^b^MMSE scores and age are inclusion criteria and stratification variables; these values were recorded at screening to ensure that there were no missing values.

^c^MMSE scores range from 0 to 30; lower scores indicate poorer cognitive performance.

^d^Scores on the 13-item cognitive subscale of the ADAS (ADAS-Cog 13). Scores range from 0 to 85; higher scores indicate greater deficit.

^e^CDR-SoB; higher score indicates greater severity of dementia (range, 0−18).

^f^ADCS-ADL; lower score indicates greater severity (range, 0−52).

NPI is the total score of 12 individual domains that ranges from 0 to 144. Higher scores indicate more behavioral disturbance.

GDS is a 30-question long-form questionnaire ranging from 0 to 30, with higher scores indicating more severe depression.

COWAT is a measure of verbal fluency. Each participant score is the mean of the number of acceptable answers given.

CFT participant score is the total acceptable words named and is scored by how long participants take to complete the test. Participantsʼ time was capped at 240 seconds.

In the WDS test, participants are asked to repeat a sequence of numbers in order and in reverse. Higher scores indicate better performance.

A two-sided Student’s *t*-test was used to compare between the placebo and treatment groups except for sex, where a chi-squared test was used. An unadjusted *P* < 0.05 was considered significant.

### Primary outcome

**Fig. 2:**
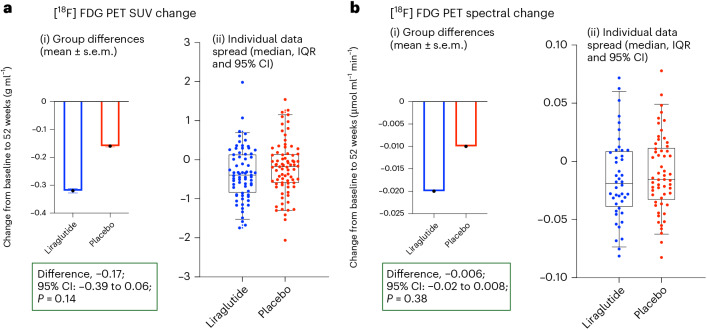
Change in primary outcome (PET SUV) and sensitivity analyses (PET spectral) at 52 weeks. (i) Figure shows change of scores at group level. Data are presented as mean ± s.e.m. (ii) Box plots show the median (center line) and interquartile range (IQR; box limits). Whiskers show the 95% confidence intervals (CIs), and points beyond this range are plotted as outliers. Individual data spread is plotted on the box-and-whiskers plot. Analysis of covariance adjusted for baseline values and stratification factors (age and MMSE) was used to compare between the placebo and treatment groups. Baseline *n* (placebo, 94; treatment, 93) and 52 weeks *n* (placebo, 82; treatment, 72) for PET SUV (**a**) and PET spectral (**b**). *P* values are unadjusted (*P* < 0.05).

No significant difference was observed between the treatment and placebo groups for the primary outcome (change in fluorodeoxyglucose ([^18^F] FDG) standard uptake value (SUV)) adjusting for baseline SUV, age and MMSE (adjusted difference = −0.17; 95% confidence interval: −0.39 to 0.06; *P* = 0.14; Fig. 2 and Supplementary Table 3). The sensitivity analysis of the spectral analysis results agreed with this conclusion.

### Secondary outcomes

**Fig. 3:**
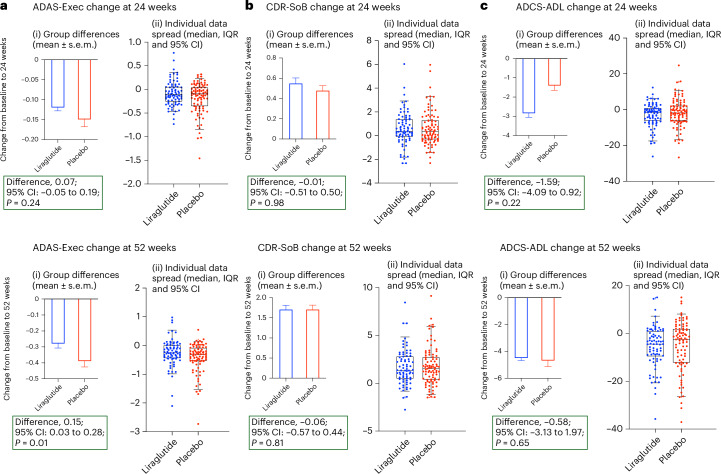
Change in key secondary outcomes—ADAS-Exec, CDR-SoB and ADCS-ADL *z*-scores at 24 weeks and 52 weeks. (i) Figure shows change of scores at group level. Data are presented as mean ± s.e.m. (ii) Box plots show the median (center line) and interquartile range (IQR; box limits). Whiskers show the 95% confidence intervals (CIs), and points beyond this range are plotted as outliers. Individual data spread is plotted on the box-and-whiskers plot. A multilevel mixed-effects model was used to compare between the placebo and liraglutide treatments. **a**, Baseline *n* (placebo, 100; treatment, 100), 24 weeks *n* (placebo, 95; treatment, 83) and 52 weeks *n* (placebo, 87; treatment, 79) for ADAS-Exec. **b**, Baseline *n* (placebo, 100; treatment, 99), 24 weeks *n* (placebo, 89; treatment, 80) and 52 weeks *n* (placebo, 88; treatment, 79) for CDR-SoB. **c**, Baseline *n* (placebo, 100; treatment, 100), 24 weeks *n* (placebo, 94; treatment, 83) and 52 weeks *n* (placebo, 89; treatment, 80) for ADCS-ADL. *P* values are unadjusted (*P* < 0.05).

The secondary outcome of cognitive function—change in ADAS-Exec (ADAS-Cognitive Subscale (ADAS-Cog) and the Executive domain scores from the Neuropsychological Test Battery (NTB)) *z*-score (Fig. 3 and Supplementary Table 4)—was slower in the treatment group compared to the placebo group (0.15; 95% confidence interval: 0.03−0.28; unadjusted *P* = 0.01). No significant differences were observed in ADCS-ADL (−0.58; 95% confidence interval: −3.13 to 1.97; *P* = 0.65) or CDR-SoB (−0.06; 95% confidence interval: −0.57 to 0.44; unadjusted *P* = 0.81) scores (Fig. 3 and Supplementary Table 4).

### Safety

The incidence and severity of treatment-emergent adverse events and clinically important changes in safety assessments was a key secondary outcome of the study. There were 991 adverse events in the 12 months of the study. There were 450 adverse events in the placebo arm occurring in 87 participants and 541 in the treatment arm occurring in 88 participants. There were 25 recorded serious adverse events in the 12 months of the study occurring in 18 (17.6%) participants on placebo and in seven (6.9%) participants on treatment. Most serious adverse events were considered unlikely to be related to the study treatment. There were 14 unexpected serious adverse events in the placebo arm and four unexpected serious adverse events in the treatment arm. There was one life-threatening serious adverse event in the placebo group. All adverse events are shown in Supplementary Table 5.

The most common adverse event was a gastrointestinal disorder, which had a higher incidence rate in the treatment group compared to the placebo group and accounted for 25.5% of all adverse events reported in those treated with liraglutide. Common gastrointestinal side effects included anorexia, bloating, diarrhea, dyspepsia, nausea and weight loss. Up to 5% weight loss was experienced by 39.2% of patients in the liraglutide arm and by 12.6% of patients in the placebo arm, and 5–10% weight loss was experienced by 8.9% of patients in the treatment arm and by 1.1% of patients in the placebo arm.

Liraglutide has a clinically acceptable safety profile for the treatment of Alzheimer’s disease syndrome and was well tolerated in participants without diabetes or obesity.

### Exploratory outcomes

**Fig. 4:**
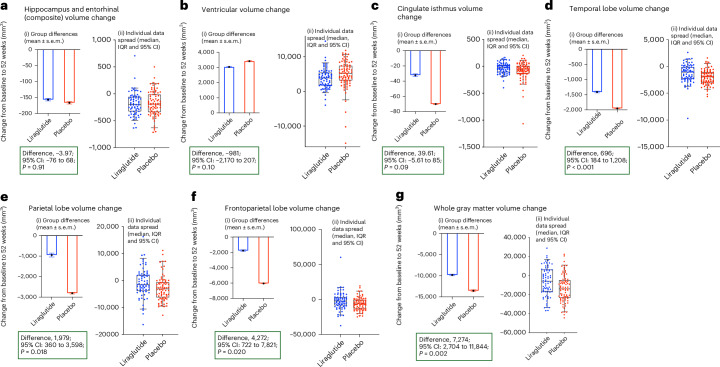
Changes in other secondary outcomes—MRI volumes at 52 weeks. MRI analyses for composite region brain (**a**), ventricular (**b**), cingulate isthmus (**c**), temporal lobe (**d**), parietal lobe (**e**), frontoparietal lobe (**f**) and whole gray matter (**g**). (i) Figure shows change of scores at group level. Data are presented as mean ± s.e.m. (ii) Box plots show the median (center line) and interquartile range (IQR; box limits). Whiskers show the 95% confidence intervals (CIs), and points beyond this range are plotted as outliers. Individual data spread is plotted on the box-and-whiskers plot. Analysis of covariance adjusted for baseline values and stratification factors (age and MMSE) was used to compare between the placebo and treatment groups. Baseline *n* (placebo, 101; treatment, 101) and 52 weeks *n* (placebo, 83; treatment, 75). *P* values are unadjusted (*P* < 0.05). A predefined *P* < 0.01 was used.

No significant treatment difference was observed on MRI volumes in hippocampus and entorhinal cortex or ventricular volume (Fig. 4 and Supplementary Table 6). However, the exploratory analyses of liraglutide-treated participants showed lower volume reductions in the temporal lobe (696 mm^3^; 95% confidence interval: 184.37−1,208.12; *P* < 0.001) and total gray matter volume (7,274 mm^3^; 95% confidence interval: 2,704.05−11,844.8; unadjusted *P* = 0.002) compared to the placebo group (Supplementary Table 6). Additionally, parietal lobe (1,978 mm^3^; 95% confidence interval: 360.12−3,597.67; unadjusted *P* = 0.018) and frontoparietal lobe (4,272 mm^3^; 95% confidence interval: 722.41−7,820.64; unadjusted *P* = 0.02) showed a trend of lower reduction in volume.

**Fig. 5:**
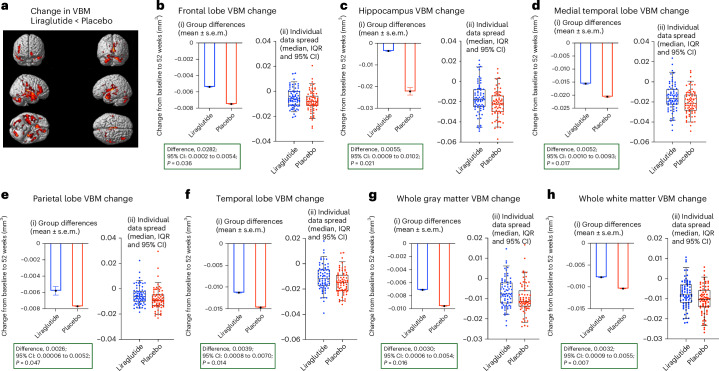
Changes in exploratory outcomes—VBM analyses at 52 weeks. Panel **a** shows reduced voxel-wise decline of gray matter VBM in participants treated with liraglutide. VBM analyses are reported for frontal lobe (**b**), hippocampus (**c**), medial temporal lobe (**d**), parietal lobe (**e**), temporal lobe (**f**), whole gray matter (**g**) and whole white matter (**h**). (i) Figure shows change of scores at 52 weeks at the group level. Data are presented as mean ± s.e.m. (ii) Box plots show the median (center line) and interquartile range (IQR; box limits). Whiskers show the 95% confidence intervals (CIs), and points beyond this range are plotted as outliers. Individual data spread is plotted on the box-and-whiskers plot. Analysis of covariance adjusted for baseline values and stratification factors (age and MMSE) was used to compare between the placebo and liraglutide treatments. Baseline *n* (placebo, 101; treatment, 101) and 52 weeks *n* (placebo, 83; treatment, 75). *P* values are unadjusted (*P* < 0.05).

Exploratory regional voxel-based morphometry (VBM) analysis (Fig. 5 and Supplementary Table 7) demonstrated that liraglutide-treated participants showed a trend of slower reduction in frontal (2.82 × 10^−3^; 95% confidence interval: 2.10 × 10^−4^ to 5.44 × 10^−3^; unadjusted *P* < 0.036), parietal lobe (2.61 × 10^−3^; 95% confidence interval: 5.9 × 10^−5^ to 5.16 × 10^−3^; unadjusted *P* < 0.047), temporal (3.90 × 10^−3^; 95% confidence interval: 8.27 × 10^−4^ to 6.98 × 10^−3^; unadjusted *P* < 0.014), whole cortical gray matter (2.98 × 10^−3^; 95% confidence interval: 5.91 × 10^−4^ to 5.36 × 10^−3^; unadjusted *P* < 0.016) and white matter (3.22 × 10^−3^; 95% confidence interval: 9.30 × 10^−4^ to 5.51 × 10^−3^; unadjusted *P* < 0.007) volumes compared to placebo-treated participants (Fig. 5 and Supplementary Table 7).

## Discussion

In this first phase 2b study, we evaluated the effect of liraglutide on glucose metabolism, cognition and MRI volume in patients with Alzheimer’s disease syndrome. Although this study revealed no significant changes in the cerebral metabolic rate of glucose in participants treated with liraglutide compared to those treated with placebo, the secondary trial outcomes revealed a beneficial treatment effect of slowing cognitive deterioration (ADAS-Exec), and liraglutide was generally safe and had an acceptable safety profile. The exploratory outcome measure revealed a beneficial effect of slowing of brain volume loss. The liraglutide-treated participants performed better on the comprehensive ADAS-Exec cognitive battery (which is a combination of ADAS-Cog and Executive domain scores from the NTB) but not on other cognitive measures (CDR-SoB or ADCS-ADL) after 52 weeks of treatment compared to the placebo group. As the results were not corrected for multiple comparisons due to the exploratory nature of the study, this must be interpreted with caution. Although the improvement started to appear before 6 months, it was more obvious by 52 weeks, suggesting that GLP-1 analogs may positively influence cognition in Alzheimer’s disease over a prolonged period of treatment, and the preserved MRI outcomes may be compatible with a possible neuroprotective effect. Due to the exploratory nature of this phase 2b study, these findings need to be confirmed in larger studies.

Preservation of cognitive function observed in liraglutide-treated participants was consistent with preclinical findings^20,21^ and the cognitive benefits identified in a meta-analysis of antidiabetic agents in participants with Alzheimer’s disease^22^. In our study, the ADAS-Exec *z*-score for cognitive function was higher for the placebo group at baseline; the slope of decline changed at 24 weeks and became significant at 52 weeks (Fig. 2). This may be due to liraglutide exerting its effect via neuroprotective mechanisms rather than having a direct symptomatic efficacy, which would have been evident within 24 weeks. The symptomatic agent donepezil showed cognitive improvement of Alzheimer’s disease cases by week 12, which continued at weeks 18 and 24 (ref. ^23^). Our observations are consistent with and supported by the REWIND trial, where long-term dulaglutide treatment prevented cognitive decline in participants with diabetes assessed with the Montreal Cognitive Assessment and the Digit Symbol Substitution Test^24^. Taken together with our current trial, we suggest that a neuroprotective effect may be a class effect of GLP-1 analogs.

Although we acknowledge that this was a 12-month-duration study, which may be insufficient to definitively establish long-term clinical benefit in Alzheimer’s disease, it can still yield meaningful insights, and further studies are necessary to fully establish the effectiveness of this drug. Modest cognitive or biomarker effects over this timeframe may represent early indicators of a slowed neurodegenerative process, which, if sustained, could translate into tangible long-term benefits. Determining what constitutes a clinically meaningful change in Alzheimer’s disease trials is inherently complex. This is due to the heterogeneity of disease progression, the limitations of traditional cognitive and functional scales and the often-subtle nature of early-stage change. Importantly, cognitive changes may precede observable functional or global benefit, particularly over short trial durations. Furthermore, the field lacks a universally accepted quantitative threshold for meaningfulness; rather, regulatory and clinical interpretations increasingly emphasize consistency across multiple domains, biological plausibility and the potential for preservation of autonomy and quality of life over time.

The observed attenuation of gray matter loss and reduced temporal lobe atrophy in liraglutide-treated participants aligns with evidence from other therapeutic trials, including donepezil and blarcamesine, that have shown similar effects^25,26^. However, this remains a noteworthy finding, given that such structural preservation is still relatively uncommon across the broader landscape of Alzheimer’s disease trials.

Blarcamesine demonstrated an attenuation in global brain volume loss measured by MRI and reduction of the expansion of the lateral ventricular volume compared to placebo. Volumetric MRI improvements associated with blarcamesine appeared global and may be in response to restoration of cellular homeostasis. The global improvements in volumetric MRI associated with blarcamesine are accompanied by reducing the decline of clinical disease progression, which suggests that the drug effects might be exerted by mitigating neurodegeneration^25^. In another trial, a 45% reduction of rate of hippocampal atrophy was observed in prodromal Alzheimer’s disease after 1 year of treatment with donepezil compared to placebo^26^. No significant difference was observed in neuropsychological performance between treatment groups^25^. Longer observation periods and longitudinal studies are warranted to evaluate the association between reduced rate of hippocampal atrophy and protective effects on cognition, such as memory and other clinically relevant domains. Similarly, there is a need to replicate the results in prodromal Alzheimer’s disease to understand the basic mechanism through which liraglutide impacts morphology and/or structure of brain regions affected by Alzheimer’s disease.

It is possible that MRI outcomes could be considered as a composite measure reflecting an improvement of several underlying pathological processes occurring in Alzheimer’s disease. The potential beneficial neuronal effect of liraglutide is supported by previous observations of improved intrinsic connectivity compared to placebo in individuals at risk of Alzheimer’s disease^11^. Therefore, it is possible that GLP-1 analogs, as a treatment for Alzheimer’s disease, produce observable neuroprotective effects on both brain structure and function, thus reducing the decline in participants’ cognitive function. It has been shown that liraglutide reduces neuroinflammation, reduces tau phosphorylation mediated by PI3K/Akt/GSK3β signaling^27^, attenuates toxic protein buildup and improves synaptic function in transgenic animal models of Alzheimer’s disease, all of which could decrease gray matter atrophy and ultimately provide protection against cognitive decline. It is likely that the effect of GLP-1 analogs is not region specific and influences the whole of the brain. Although we did not observe the preservation of volume in some of the smaller structures, it is likely that we would need a larger number of participants to demonstrate an effect in the smaller structures due to significant variability in the atrophy in these structures for the advanced participants who were included in the study.

Additionally, based on our cortical VBM results, the spatial distribution of significant voxels in the cortex closely resembles the spatial distribution of the expression of insulin receptor substrate 1 (IRS-1) and other molecules in the insulin signaling cascade^28^. IRS-1 is an effector molecule of the insulin receptor. Specific changes in its phosphorylation result in impaired insulin signaling, which is an established pathological marker of insulin resistance in peripheral tissues. Various differentially phosphorylated forms of IRS-1 have been considered pathological markers of insulin resistance in Alzheimer’s disease^28,29^. Impaired insulin signaling and IRS-1 signaling have been observed in the postmortem Alzheimer’s disease brain^30^. This is an indirect finding and provides evidence suggestive of target engagement by liraglutide. Liraglutide treatment has been shown to ameliorate insulin resistance aberrations and decrease IRS-1 pS^616^ upregulation in mouse models of Alzheimer’s disease^31^. This warrants additional investigation of the therapeutic potential of liraglutide and GLP-1 agonists in Alzheimer’s disease.

In the present study, we did not demonstrate a reduced change in cerebral glucose metabolism in participants treated with liraglutide. This finding differs from a previous 26-week pilot study in which liraglutide prevented a decline in glucose metabolism compared to placebo^10^. There are several possibilities to explain this discrepancy. First, failure to observe any changes in cerebral glucose metabolism probably reflects key differences in methodology. The participants continued treatment up to the scanning date in the previous study but discontinued treatment 3 days before the week 52 PET scan was acquired in the ELAD study. The rationale for conducting the scans 3 days after the final dose was to minimize the potential for acute pharmacodynamic effects (giving a minimum of five half-lives of liraglutide)—particularly those that could transiently alter systemic glucose availability or uptake mechanisms unrelated to central neuronal activity. [^18^F] FDG uptake in the brain is largely insulin independent, especially in neurons, and is thought to reflect neuronal metabolic activity rather than peripheral insulin action^32^. Although liraglutide can influence peripheral glucose metabolism, the brain’s glucose utilization, as measured by [^18^F] FDG PET, is relatively stable and less subject to short-term fluctuations in insulin sensitivity. Future studies should evaluate the influence of suddenly stopping the GLP-1 analogs on the brain after daily injections for a year. A greater number of participants may be needed to demonstrate a group difference upon treatment cessation. Second, it is possible that the wider clinical range of Alzheimer’s disease participants (MMSE scores 15−30) included in this trial may have diluted any [^18^F] FDG changes. Participants with MMSE scores lower than 18 have severe hypometabolism that is associated with advanced disease, which may be difficult to protect with liraglutide owing to the extent of neuronal damage. Third, the number of participants who completed the trial was less than that anticipated by our power calculation. Finally, levels of microglial activation may drive [^18^F] FDG signal alterations. It is also possible that as liraglutide has been shown to reduce microglial activation^33^, [^18^F] FDG signal discrepancies could also reflect that 52 weeks of treatment may also be attenuating the inflammatory response, resulting in a relative reduction in glucose metabolism in patients who received treatment.

Liraglutide was well tolerated by the participants with Alzheimer’s disease. The total number of adverse events was greater in the patient group treated with liraglutide, consistent with the side effect profile of the drug; however, serious adverse events were more common in the placebo group. Gastrointestinal adverse events were the most frequently reported in participants receiving liraglutide, an established class effect of GLP-1 receptor agonist compounds that declines over the course of treatment^34^. Once-daily injections can be a substantial burden for participants. An oral formulation of GLP-1 receptor agonist is available and regularly used in the treatment of diabetes and obesity, which would reduce this burden and benefit the level of treatment compliance in future studies, although our study did demonstrate that even daily injections in the context of a clinical trial in Alzheimer’s disease are possible.

Together, our study provides, to our knowledge, the first large-scale evaluation of GLP-1 analogs in people living with Alzheimer’s disease and provides an insight into the mechanism of action of GLP-1 analogs in neurodegenerative diseases. There is an unmet need to identify effective treatment strategies beyond targeting β-amyloid pathology. Although promising, novel anti-β-amyloid therapies provide only modest improvements to activities of daily living and cognitive benefits despite effectively reducing β-amyloid load. Alzheimer’s pathogenesis is multifaceted, and here we indicate a cognitive benefit of a candidate that is evidenced to influence several pathological aspects of Alzheimer’s disease beyond β-amyloid. This is a notable finding, demonstrating the ability of non-β-amyloid targets to provide cognitive benefit and neuroprotection and showing the importance of developing alternate treatments to accompany therapies such as lecanemab and, potentially, donanemab. To identify an effective treatment strategy, we indicate that a multitargeted approach is essential for the future of Alzheimer’s therapy. With a well-established safety profile in patients with obesity and diabetes, we demonstrate that liraglutide is generally safe and well tolerated in this neurodegenerative population.

Participants were diagnosed in specialized centers after detailed clinical and neurological examination, neuropsychometric evaluation and MRI scans. All participants had repeat MRI and [^18^F] FDG PET as a part of the study. Any patient whose MRI and [^18^F] FDG PET were not consistent with a diagnosis of Alzheimer’s disease was excluded.

Although β-amyloid PET, tau PET or translocator protein (TSPO) PET were outcome measures, owing to participants’ burden due to the number of PET scans and that these scans were optional, participants did not proceed to have the optional β-amyloid PET, tau PET or TSPO PET, and we were unable to evaluate regional levels of β-amyloid, tau deposition and neuroinflammation. We collected blood samples of patients at baseline and follow-up; however, the plasma biomarkers have not been analyzed. As a result, we cannot provide complementary biomarker evidence to contextualize the observed metabolic findings. Future studies incorporating both β-amyloid biomarkers and genetic risk stratification (APOE genotype) will be important to fully understand treatment heterogeneity. Moreover, integrating blood-based and imaging-based biomarkers alongside genetic profiling will better elucidate mechanisms and treatment effects.

A key limitation of this study was that attrition reduced the achieved sample size compared to that planned in the original power calculation. Although power calculations are prospective tools and are not typically reestimated post hoc, the reduced sample size likely lowered the probability of detecting small effects and limits replication confidence. In addition, no formal power calculation was performed for secondary or exploratory endpoints; analyses should, therefore, be interpreted as exploratory and hypothesis generating. Nonetheless, the study successfully demonstrated feasibility, safety and some early biological signals.

Moreover, a key limitation of the present study was the limited population diversity, in that most of the participants were White. The ethnic composition of the population makes it challenging to assess whether findings are representative across different demographic groups and generalizable to the general public. In addition, important sources of heterogeneity, such as diet and medical history, were not described or modeled, limiting the ability to account for potential confounders that may influence treatment response. However, participantsʼ medical history that may influence cognitive function was not included in the study.

We performed mean imputation that has limitations and may not fully preserve variability or multivariate relationships. Only four patients had missing data at baseline, which makes up 2% of the study population. Hence, mean imputation in the present study was applied only to baseline covariates. More sophisticated approaches, such as multiple imputation, would be preferable in larger confirmatory trials.

The ELAD study showed no significant changes in cerebral glucose metabolism in participants with mild to moderate Alzheimer’s disease syndrome, which was the primary outcome measure. The secondary outcome measures revealed that patients treated with liraglutide had a significantly slower decline in cognition (ADAS-Exec) and a slower reduction in MRI brain volume compared to the placebo arm, demonstrating a favorable response to liraglutide treatment. Liraglutide was well tolerated by patients with Alzheimer’s disease syndrome.

## Methods

### Study protocol and population

Between March 2014 and January 2021, we conducted a 12-month, multicenter, randomized, double-blind, placebo-controlled phase 2b trial in 24 sites across the UK. All PET and MRI scanning was performed at a single site at the Imperial College Clinical Imaging Facility, and other trial-related activities were performed at the individual sites. Participants were recruited from memory clinics and Dementia and Neurodegenerative Diseases Research Networks.

Eligible participants were aged 50 years or older with a clinical diagnosis of mild to moderate Alzheimer’s disease based on detailed clinical and neurological examination, neuropsychometric evaluation and MRI scans in the secondary or tertiary centers as defined by the National Institute of Neurological and Communicative Diseases and Stroke/Alzheimer’s Disease and Related Disorders Association. Participants with Alzheimer’s disease had an MMSE score of 15 or higher and a CDR Global score of 0.5, 1 or 2, calculated using the University of Washington online algorithm. Participants who were taking treatment for diabetes mellitus were excluded. A complete list of the inclusion and exclusion criteria is provided in the supplementary files.

This study was approved by the local and regional Regulatory Ethics Committees (National Research Ethics Committee-Riverside and Imperial College London/Imperial College Healthcare NHS Trust Joint Research Office) and the Medicines and Healthcare products Regulatory Agency (MHRA). Approval for the administration of radioactivity was given by the Administration of Radioactive Substances Advisory Committee. Written informed consent was provided by all participants before participation in the trial. An Independent Data Monitoring Committee and an Independent Trial Steering Committee met regularly during the trial to monitor the safety and conduct of the trial. Clinical data will be entered directly into computers via Inform. The data manager will arrange appropriate quality assurance checks. After each assessment, data will be entered in the study database. Participants will be identified by their unique patient identifier only. The trial protocol and statistical analysis plan are provided in the supplementary materials.

### Randomization and masking

A total of 204 participants were randomized to receive the active drug or placebo with a 1:1 allocation ratio using stratified block randomization with a fixed block size of six. Randomization was performed by Mawdsley and Brooks. Participants were stratified according to age groups (age 50–75 years and age >75 years) and MMSE scores (MMSE 15–24 and MMSE >24).

### Intervention

In animal models of Alzheimer’s disease, a liraglutide dose of 0.25 mg kg^−1^ d^−1^ was used to test its efficacy, which translates to a human equivalent dose of 1.2−1.8 mg of liraglutide daily in a 60−90-kg human, as per the US Food and Drug Administration (FDA) conversion table 2005 (https://www.fda.gov/media/72309/download)^35^. In humans, liraglutide at doses up to 1.8 mg has been approved in several countries, including the European Union, Japan, Australia and the United States, for the treatment of type 2 diabetes under the trade name Victoza. In March 2015, the European Medicines Agency (EMA) approved its use in obesity under the trade name Saxenda, at doses up to 3 mg. The doses used in the ELAD trial are those approved for diabetes, as, at the time of the study design, liraglutide was only approved for clinical use in diabetes^36^.

Pharmacokinetic data from the clinical development program for liraglutide demonstrated that it is absorbed slowly (tmax = 8–12 hours) and has a half-life of approximately 13 hours^37^. Although liraglutide’s brain penetration is limited, pharmacokinetic and functional data demonstrate that sufficient concentrations of liraglutide reach the central nervous system (CNS) to activate GLP-1 receptors, including in cortical and hypothalamic neurons^37^. Preclinical (animal model) studies have demonstrated that liraglutide suppresses β-amyloid accumulation and tau hyperphosphorylation and inflammation^38,39^. Thus, liraglutide does pharmacokinetically engage CNS targets at therapeutic doses. Liraglutide is suitable for once-daily subcutaneous injection given any time of the day, independent of meals. Investigation of liraglutide metabolism in vitro and in healthy participants has indicated that liraglutide is endogenously metabolized and that neither renal excretion nor hepatic extraction is a major route of clearance. The pharmacokinetics of liraglutide has been investigated in human participants with renal and hepatic impairment and has not raised any safety concerns. However, the therapeutic experience in participants with hepatic or renal impairment is limited. The effects of age and gender on the pharmacokinetics of liraglutide have been investigated, and it was concluded that all participants, regardless of age or gender, should be dosed in accordance with the usual proposed dose regimen for liraglutide^36^.

Participants received once-daily injections of liraglutide or placebo. The study drug was administered as a daily subcutaneous injection, commencing with a dose of 0.6 mg once daily, and the dose was escalated to 1.8 mg within 4 weeks. Participants who did not tolerate 1.8 mg remained on 1.2 mg for an additional 2 weeks, and then two more attempts were made to increase the dose to 1.8 mg. If participants did not tolerate 1.2 mg, they were withdrawn from the study but were included in the safety reporting. After completion of the 52-week treatment period, participants were given the opportunity to participate in a 12-month open-label extension, in which they received liraglutide, the results of which are not reported here.

There were no recorded treatment crossovers in this trial. The compliance was measured in two ways: the total drug prescribed to individuals, dispensed at visits and returned at visits (measured in mg), and the number of injections taken according to diaries and time on the trial (participants provided diary information at each visit). The mg prescribed compliance was calculated according to:

$$\begin{array}{l}\mathrm{mg}\,\mathrm{prescribed}\,\mathrm{and}\,\mathrm{returned}\,\mathrm{compliance}\,\left( \% \right)\\ =\frac{\mathrm{Total}\,\mathrm{mg}\,\mathrm{dispensed}-\mathrm{Total}\,\mathrm{mg}\,\mathrm{left}\,\mathrm{over}}{\mathrm{Total}\,\mathrm{mg}\,\mathrm{prescribed}}\times 100\end{array}$$

The number of injections taken compliance was calculated according to:

$$\begin{array}{l}\mathrm{Number}\,\mathrm{of}\,\mathrm{injections}\,\mathrm{taken}\,\left( \% \right)\\ =\frac{\mathrm{Total}\,\mathrm{injections}\,\mathrm{taken}\,\mathrm{according}\,\mathrm{to}\,\mathrm{diaries}}{\mathrm{Days}\,\mathrm{on}\,\mathrm{trial}}\times 100\end{array}$$

### Data collection

The patients were recruited from memory clinics across the UK in 24 sites. All patients had a diagnosis of Alzheimer’s disease based on their clinical evaluation, neurological examination, neuropsychometric evaluation and MRI scans. All patients were provided with a detailed patient information sheet with adequate time to consider the study. Participants were then invited to the trial site, and written informed consent was obtained. All participants underwent a detailed screening test that involved reviewing the clinical history and diagnosis, collecting medical history and ensuring that the patient satisfied the inclusion/exclusion criteria. Participants had detailed physical and neurological examination and neuropsychometric evaluation. Patients also underwent vital signs, 12-lead electrocardiogram (ECG), laboratory tests and safety blood tests. Patients had 17 visits in total during the 52-week study. After the screening, eligible participants underwent a baseline visit, as detailed in the supplementary materials. Participants were given liraglutide 1.8 mg per day or placebo. Safety visits were performed at weeks 0, 1, 4, 8, 12, 16, 20, 24, 28, 32, 36, 40, 44, 48, 52 and 56. [^18^F] FDG and MRI scans were completed at baseline and at week 52 visits. ADAS-Exec, CDR-SoB and ADCS-ADL were rated at baseline, week 24 and week 52.

### Outcomes

#### Neuroimaging measures

The primary outcome measure was the change in rCMRglc in the cortical regions (combination of regions: hippocampal, medial temporal lobe and posterior cingulate) from baseline to follow-up (12 months) in the treatment group compared to the placebo group. rCMRGlc was estimated by creating parametric maps using spectral analysis with an arterial plasma input function as previously described^40,41^. We used a lumped constant of 0.48. Additionally, SUV was calculated using the following formula: $\frac{\mathrm{Tracer}\,\mathrm{uptake}\,\mathrm{value}}{(\mathrm{Dose}\,\mathrm{of}\,\mathrm{radioactivity}\,\mathrm{injected}/\mathrm{Body}\,\mathrm{weight})}$.

A region of interest analysis was undertaken for rCMRGlc and SUV images, and all individual images were co-registered to their corresponding MRIs. Object maps were created by multiplying the binarized MRI with the probabilistic atlas using SPM8 in MRI space to create individualized object maps of volumes of interest. Parametric maps were then sampled combining hippocampus, medial temporal lobe and posterior cingulate cortex using Analyze AVW. We assessed cerebral glucose metabolism in a composite region combining the hippocampus, medial temporal lobe and posterior cingulate cortex from rCMRglc and SUV parametric images of [^18^F] FDG PET scans.

The key secondary outcomes included a change from baseline to 12 months in *z*-scores for the ADAS-Exec (ADAS-Cog and the Executive domain scores from the NTB), the incidence and severity of treatment-emergent adverse events or clinically important changes in safety assessments over 12 months. CDR-SoB and ADCS-ADL were also secondary outcome measures.

MRI changes in the volume of the ventricles and a composite region (hippocampus and entorhinal cortex) from baseline to 52 weeks were assessed in a predefined secondary analysis. Participants’ T1 MRI volumetric scans at baseline and 52 weeks were processed using FreeSurfer software 7.4.1 (ref. ^42^). Predefined exploratory analysis was performed for temporal lobe, ventricles and isthmus cingulate. As GLP-1 analogs can have global effects, we performed additional analyses for whole gray matter, frontoparietal and occipital lobe volumes. (Image processing of [^18^F] FDG PET imaging and MRI scans are detailed in the supplementary materials.)

VBM analysis was performed as an exploratory analysis of brain macrostructure at a regional and voxel-wise level. Regional analysis was conducted within the hippocampus, medial temporal lobe, anterior cingulate, posterior cingulate, frontal lobe, parietal lobe, occipital lobe, temporal lobe volume, whole gray matter and whole white matter.

Safety and tolerability were assessed based on all reported adverse events and serious adverse events. Clinically significant abnormalities in vital signs, laboratory evaluations, ECG recordings and physical examinations were recorded as adverse events on the relevant medical/psychiatry history. Key secondary outcomes included the change from baseline to 52 weeks in *z*-scores for the ADAS-Exec, CDR SoB and ADCS-ADL.

The ADAS-Cog has been employed as a cognitive efficacy measure in most Alzheimer’s disease clinical drug trials. However, its use has been criticized on the grounds that it does not index all the functions known to be compromised early on in Alzheimer’s disease, and, because measurement is variable, studies require many study participants. Additional tests have been added to remedy the deficiencies of the original instrument^43^. However, it is not clear that they have successfully remedied the identified issues. Alternative instruments, such as the NTB^44^, have been validated and accepted for use. However, even when used in tandem with the ADAS-Cog, the NTB does not satisfactorily map all the cognitive domains specified by the EMEA^44^. To remedy these issues, we combined the standard 13-item ADAS-Cog with the executive function components of the NTB (namely, Controlled Oral Word Association Test (COWAT), Category Fluency Test (CFT), Wechsler Digit Span (WDS) and Trail Making Test (TMT)). Performance on the ADAS-Exec is summarized as a composite to which each measure contributes equally via *z*-score transformation. Support for the utility of this approach was previously provided by applying this methodology to the data from proof-of-concept trials of PBT2 (refs. ^45,46^).

The total scores for ADAS-Exec were calculated for each test all in the same direction (higher score means better performance). The ADAS-Cog 13 subtest scoring (1.1−1.14) and the NTB scoring (1.15−1.18) are shown below:

- 1.1 Word recall
  The total of the correctly recalled names of word (recall1 + wordrecall2 + wordrecall3)
- 1.2 Delayed recall
  The number of words correctly recalled
- 1.3 Naming task
  Total of correctly named items (maximum 17)
- 1.4 Commands
  Total of correct commands (maximum 5)
- 1.5 Constructional praxis
  Total of correct drawings (maximum 4)
- 1.6 Ideational praxis
  Total of correct responses given (maximum 5)
- 1.7 Orientation
  Total of correct responses given (maximum 8)
- 1.8 Word recognition
  Total number of correct responses (capped at 12)
- 1.9 Language score
  Score between 0 and 5 given by examiner (higher score means better performance)
- 1.10 Comprehension
  Score between 0 and 5 given by examiner (higher score means better performance)
- 1.11 Word finding
  Score between 0 and 5 given by examiner (higher score means better performance)
- 1.12 Remembering test instructions
  Score between 0 and 5 given by examiner (higher score means better performance)
- 1.13 Mazes
  Difference between the time limit of 240 seconds and the number of seconds on the test
- 1.14 Number of cancellations
  Difference between the number of correct targets crossed off and the number of incorrect targets crossed off
- 1.15 COWAT
  Total acceptable named words regardless if missing score for one letter
- 1.16 CFT
  Total acceptable named answers
- 1.17 WDS
  - Forward total at each timepoint
  - Backward total at each timepoint
- 1.18 TMT

- Trail A: difference between the time limit of 240 seconds (maximum time limit) and the number of seconds on the test
- Trail B: difference between the time limit of 240 seconds (maximum time limit) and the number of seconds on the test

The ADAS-Exec *z*-score was calculated in the following steps:

- The screening and baseline total scores were averaged and used as a single baseline assessment. Where only one screening or baseline score was available, this value was used as the baseline.
- The mean and standard deviation were calculated for each total score at baseline.
- The participants’ *z*-score for each test was calculated at each timepoint:
  $$\begin{array}{l}z{-}{\mathrm{score}}_{t}\\ =\frac{{\mathrm{score}}_{t}-\mathrm{mean}\,{\mathrm{score}}_{\mathrm{baseline}}}{{\rm{s}}.{\rm{d}}{.}_{\mathrm{baseline}}},\mathrm{where}\,t=\mathrm{baseline},\,24\,\mathrm{weeks}\,\mathrm{and}\,52\,\mathrm{weeks}\end{array}$$
- The average *z*-score for each participant was calculated.
- ADAS for each patient was calculated as the average of the *z*-scores.
- The *z*-scores were estimated for participants with at least one NTB subtest and at least two-thirds (9/14) of the ADAS-Cog subtests completed.

Additional secondary outcomes included TSPO and tau PET, amyloid load and APOE4 status. Patients were keen to undergo minimal PET imaging; therefore, tau and TSPO PET were not completed as part of this study, and cerebrospinal fluid data were also not collected. Genotyping and plasma markers have not yet been analyzed and are not reported in the paper.

### Statistical analysis

Landau et al.^47^ used the [^18^F] FDG PET imaging biomarker to monitor the progression of Alzheimer’s disease. At and after 12 months, they found a mean change in the [^18^F] FDG region of interest of −0.055, with an s.d. of 0.068 (ref. ^47^). Assuming that the treatment reduces the mean change of [^18^F]FDG SUV in the participants with Alzheimer’s disease to −0.025 (44% effect size), 82 participants would be required per group to provide 80% power at a 5% significance level. Allowing for a dropout of 15% over the study period, the trial required 103 participants per group (206 in total). Statistical analyses were performed using MATLAB version 2020a, and all *P* values were two-sided. A significance level of 0.05 was used to declare statistical significance for the primary outcome, key secondary outcomes and other secondary outcomes. A predefined significance level of 0.01 was used to declare statistical significance for any exploratory analyses. 95% confidence intervals are reported throughout. In this study, only the primary endpoint was designated for confirmatory testing and subjected to the predefined type I error control. All secondary and exploratory endpoints were analyzed to generate hypotheses for future trials.

The primary analysis was intention to treat and involved all patients who were randomly assigned. An analysis of covariance was undertaken for the primary outcome of participants who completed the baseline and follow-up scans, with rCMRglc as the response variable and adjusting for stratification factors (age and MMSE) and baseline values of rCMRglc. No missing data were imputed for the outcome data, and any missing baseline data were imputed as the mean of the participants with this measurement^48^ (irrespective of treatment). We calculated the adjusted mean difference in change in rCMRglc in the composite cortical region between randomized groups at 12 months with 95% confidence interval and associated two-sided *P* value. The primary outcome was analyzed using two methods: SUV and spectral analysis. We had 101 participants for the analysis using spectral analysis with arterial plasma input, whereas the less invasive SUV method had 154 participants with baseline and 52-week analyzable scans. The SUV results were analyzed as the primary outcome, and the spectral analyses were analyzed with the same method as part of the sensitivity analysis.

A multilevel mixed-effects model was used to interrogate the repeated key secondary outcome measures at 24 weeks and 52 weeks. The model allowed for an interaction between treatment and time as a categorical variable and was adjusted for the randomization factors and baseline values of the outcome. An analysis of covariance as described for the primary outcome (without imputation) was used for the MRI volumetric secondary and exploratory outcomes. A change in volume from baseline to 52 weeks was analyzed as part of an exploratory analysis. *P* < 0.01 was considered significant.

Safety and tolerability assessments included the monitoring and recording of all adverse events and serious adverse events as well as the regular monitoring of vital signs. Clinically significant abnormalities in vital signs, laboratory evaluations, ECG recordings and physical examinations were recorded as adverse events and followed-up as appropriate. Adverse events were described by duration, severity grade, relationship to the study drug, the actions taken and the outcome if relevant.

All analysis was conducted using Stata version 15.1 (StataCorp LLC).

### Patient and public involvement

During the design and conduct phase of this study, participants from the Alzheimer’s Society (patient representative groups) reviewed the study proposal and all study-related materials. They considered this study highly important and strongly supported it. Their input was incorporated into the study protocol, patient information sheet and other study-related materials. Throughout the study, the Patient and Public Involvement group met regularly and provided advice on improving recruitment and on other challenges met during the study.

### Reporting summary

Further information on research design is available in the Nature Portfolio Reporting Summary linked to this article.

## Online content

Any methods, additional references, Nature Portfolio reporting summaries, source data, extended data, supplementary information, acknowledgements, peer review information; details of author contributions and competing interests; and statements of data and code availability are available at 10.1038/s41591-025-04106-7.

## Supplementary information

Supplementary Information Supplementary Tables 1–7.

Reporting Summary

## Data Availability

The deidentified individual participant data that underlie the results reported in this paper (including text, tables and figures) are included in the paper and supplementary data files. Source data are provided with this paper. All data generated or analyzed during this study are included in this published article (and its Supplementary Information files).
