# Supplementary material for: Liraglutide in mild to moderate Alzheimer’s disease: a phase 2b clinical trial
Source: Nat Med. 2025 Dec 1;32(1):353–61. doi: 10.1038/s41591-025-04106-7 (PMC12823385; doi:10.1038/s41591-025-04106-7)
Supplement: Supplementary file 1 — Supplementary Tables 1–7. [file 41591_2025_4106_MOESM1_ESM.pdf]

nature medicine

Supplementary information https://doi.org/10.1038/s41591-025-04106-7

Liraglutide in mild to moderate Alzheimer's disease: a phase 2b clinical trial

In the format provided by the authors and unedited

Supplementary

Supplementary Table 1. Proportions of patients with MMSE score <18.

	Placebo	Treatment	Total
MMSE ≤18	13	10	23
MMSE >18	89	92	181
Total	102	102	204

Supplementary Table 2. Proportions of patients with CDR scores of 0, 0.5 and 1.

	Placebo	Treatment	Total
CDR = 0.5	60	61	121
CDR = 1	38	40	78
CDR = 2	2	1	3
Total	100	102	202

Supplementary Table 3. Primary outcomes in ELAD[a].

**Variable**	**Visit**	**Placebo n = 102**	**Treatment n = 102**	**Between-group difference (95% CI)**	**p-value**
SUV (g/ml)[b,c]	Baseline	5.44±1.01	5.43±0.92		
	52 weeks	5.28±1.04	5.11±1.00	-0.17 (-0.39, 0.06)	0.14
Spectral analysis (µmol/ml/min)[d]	Baseline	0.24±0.04	0.25±0.04		
	52 weeks	0.23±0.04	0.23±0.04	-0.006 (-0.02, 0.008)	0.38

a—Referring to the participants with data at each time-point (for baseline results, this is before imputation if applicable).

b—ANCOVA model adjusted for randomisation factors (age and MMSE as continuous variables) and baseline values of the outcomes. Missing baseline data impute for primary analysis and sensitivity analysis only. Missing outcome data imputed only for sensitivity analysis of the primary outcome.

c—Baseline SUV values imputed as the overall mean regardless of treatment.

d—Please note, the spectral analysis is reported as a rate and a much smaller scale (μmol/ml/min) than the SUV scale (g/ml).

A significance level of 0.05 was used to declare statistical significance. 95% confidence intervals (CI) are reported throughout.

Supplementary Table 4. Key secondary outcomes in ELAD[a].

Variable	Visit	Placebo n = 102	Treatment n = 102	Between-group difference (95% CI)	p-value
ADAS-Exec[b]	Baseline	0.01±0.55	-0.03±0.56		
	24 weeks	-0.14±0.73	-0.15±0.64	0.07 (-0.05, 0.19)	0.24
	52 weeks	-0.38±0.91	-0.31±0.83	0.15 (0.03, 0.28)	0.01
CDR-SoB[b]	Baseline	3.64±1.72	3.67±1.81		
	24 weeks	4.12±2.18	4.22±2.34	-0.01 (-0.51, 0.50)	0.98
	52 weeks	5.35±2.80	5.18±2.85	-0.06 (-0.57, 0.44)	0.81
ADCS-ADL[b]	Baseline	66.07±9.65	66.51±9.46		
	24 weeks	64.66±12.10	63.66±11.49	-1.59 (-4.09, 0.92)	0.22
	52 weeks	61.37±13.99	62.00±11.19	-0.58 (-3.13, 1.97)	0.65

a—Referring to the participants with data at each time-point (for baseline results, this is before imputation if applicable).

b—Key secondary outcomes treatment effects were obtained from a multilevel mixed-effects model including repeated measures of the secondary outcome variables at the relevant time points post randomisation (level 1) nested within participants (level 2). The model was adjusted for randomisation factors (age and MMSE as continuous variables) and baseline values of the outcomes.

A significance level of 0.05 was used to declare statistical significance. 95% confidence intervals (CI) are reported throughout.

Supplementary Table 5. Adverse Events by CTCAE.

CTCAE	Treatment (N=102)		Placebo (N=102)		P-value
	n	%	n	%	
Abdominal pain	3	2.9	5	4.9	0.7211
Agitation	2	1.9	1	1	>0.9999
Alanine aminotransferase increased	0	0	1	1	>0.9999
Allergic rhinitis	1	1	2	2	>0.9999
Alopecia	1	1	0	0	>0.9999
Anemia	4	3.9	2	2	0.6828
Anorexia	26	25.2	4	3.9	<0.0001
Anxiety	3	2.9	4	3.9	>0.9999
Arthralgia	2	1.9	4	3.9	0.6828
Arthritis	0	0	6	5.9	0.0290
Atrial fibrillation	1	1	2	2	>0.9999
Atrial flutter	0	0	1	1	>0.9999
Atrioventricular block first degree	0	0	1	1	>0.9999
Back pain	5	4.9	9	8.8	0.4071
Belching	1	1	0	0	>0.9999
Bladder infection	2	1.9	0	0	0.4975
Bloating	4	3.9	0	0	0.1213
Blood bilirubin increased	0	0	1	1	>0.9999
Blurred vision	0	0	1	1	>0.9999
Bronchial infection	0	0	1	1	>0.9999
Bruising	0	0	1	1	>0.9999
Burn	1	1	0	0	>0.9999
Cardiac disorders	1	1	1	1	>0.9999
Chest pain - cardiac	0	0	2	2	0.4975
Chest wall pain	1	1	0	0	>0.9999
Cholesterol high	1	1	1	1	>0.9999
Chronic kidney disease	2	1.9	0	0	0.4975
Conduction disorder	2	1.9	0	0	0.4975
Confusion	3	2.9	5	4.9	0.7211
Conjunctivitis	0	0	1	1	>0.9999
Constipation	13	12.6	3	2.9	0.0166
Cough	6	5.8	15	14.7	0.0632
Creatinine increased	0	0	2	2	0.4975
Dehydration	1	1	0	0	>0.9999
Delirium	1	1	0	0	>0.9999
Delusions	0	0	1	1	>0.9999
Depression	6	5.8	6	5.9	>0.9999

Diarrhea	17	16.5	10	9.8	0.2146
Dizziness	7	6.8	12	11.8	0.3355
Dry eye	0	0	2	2	0.4975
Dry mouth	1	1	0	0	>0.9999
Dry skin	0	0	3	2.9	0.2463
Dysgeusia	1	1	0	0	>0.9999
Dyspepsia	10	9.7	4	3.9	0.1643
Dyspnea	0	0	1	1	>0.9999
Eczema	1	1	1	1	>0.9999
Edema limbs	0	0	2	2	0.4975
Enterocolitis	2	1.9	0	0	0.4975
Epistaxis	2	1.9	1	1	>0.9999
Eye disorders	0	0	1	1	>0.9999
Eye infection	1	1	1	1	>0.9999
Fall	6	5.8	6	5.9	>0.9999
Fatigue	13	12.6	12	11.8	>0.9999
Fecal incontinence	1	1	0	0	>0.9999
Flatulence	1	1	0	0	>0.9999
Flu like symptoms	0	0	1	1	>0.9999
Flushing	1	1	0	0	>0.9999
Folliculitis	0	0	1	1	>0.9999
Fracture	1	1	0	0	>0.9999
Gallbladder pain	0	0	1	1	>0.9999
Gastritis	1	1	1	1	>0.9999
Gastroesophageal reflux disease	1	1	0	0	>0.9999
Gastrointestinal disorders	1	1	2	2	>0.9999
Generalized oedema	1	1	1	1	>0.9999
Glaucoma	1	1	1	1	>0.9999
Hair texture abnormal	0	0	1	1	>0.9999
Hallucinations	2	1.9	0	0	0.4975
Headache	12	11.7	10	9.8	0.8220
Hearing impaired	0	0	2	2	0.4975
Hematoma	3	2.9	10	9.8	0.0820
Haematuria	4	3.9	3	2.9	>0.9999
Haemorrhoids	0	0	1	1	>0.9999
Hirsutism	0	0	1	1	>0.9999
Hoarseness	0	0	1	1	>0.9999
Hydrocephalus	1	1	0	0	>0.9999
Hyperglycaemia	1	1	4	3.9	0.3688
Hyperhidrosis	2	1.9	1	1	>0.9999
Hyperkalaemia	1	1	2	2	>0.9999
Hyperlipidaemia	0	0	1	1	>0.9999

Hyperparathyroidism	1	1	0	0	>0.9999
Hypertension	5	4.9	2	2	0.4449
Hyperuricemia	0	0	1	1	>0.9999
Hypoglycaemia	1	1	1	1	>0.9999
Hypokalaemia	0	0	1	1	>0.9999
Hypotension	4	3.9	4	3.9	>0.9999
Injection site reaction	5	4.9	5	4.9	>0.9999
Injury, poisoning and procedural complications	4	3.9	4	3.9	>0.9999
Insomnia	4	3.9	0	0	0.1213
Investigations	5	4.9	8	7.8	0.5682
Irritability	0	0	1	1	>0.9999
Joint effusion	1	1	0	0	>0.9999
Keratitis	1	1	0	0	>0.9999
Kidney infection	0	0	1	1	>0.9999
Laryngeal haemorrhage	0	0	1	1	>0.9999
Laryngeal inflammation	0	0	1	1	>0.9999
Lethargy	6	5.8	1	1	0.1185
Leukocytosis	1	1	0	0	>0.9999
Lipase increased	7	6.8	2	2	0.1699
Lung infection	9	8.7	12	11.8	0.6459
Lymphocyte count increased	0	0	1	1	>0.9999
Memory impairment	1	1	1	1	>0.9999
Metabolism and nutrition disorders	1	1	2	2	>0.9999
Movements involuntary	0	0	1	1	>0.9999
Mucositis oral	0	0	1	1	>0.9999
Muscle cramp	2	1.9	3	2.9	>0.9999
Muscle weakness upper limb	0	0	1	1	>0.9999
Musculoskeletal and connective tissue disorder	1	1	2	2	>0.9999
Myalgia	1	1	1	1	>0.9999
Nasal congestion	1	1	3	2.9	0.6213
Nausea	26	25.2	8	7.8	0.0012
Neck pain	3	2.9	3	2.9	>0.9999
Neoplasms benign, malignant and unspecified (incl cysts and polyps)	2	1.9	8	7.8	0.1007
Nervous system disorders	4	3.9	3	2.9	>0.9999
Non-cardiac chest pain	3	2.9	3	2.9	>0.9999
Pain in extremity	1	1	5	4.9	0.2118
Palpitations	0	0	1	1	>0.9999
Paraesthesia	2	1.9	1	1	>0.9999
Periodontal disease	2	1.9	0	0	0.4975
Personality change	1	1	0	0	>0.9999

Pharyngitis	0	0	3	2.9	0.2463
Phlebitis	1	1	0	0	>0.9999
Photosensitivity	0	0	1	1	>0.9999
Platelet count decreased	1	1	1	1	>0.9999
Postnasal drip	1	1	0	0	>0.9999
Presyncope	1	1	1	1	>0.9999
Productive cough	0	0	1	1	>0.9999
Pruritus	2	1.9	1	1	>0.9999
Psychiatric disorders	3	2.9	4	3.9	>0.9999
Rash maculo-papular	3	2.9	4	3.9	>0.9999
Renal colic	1	1	0	0	>0.9999
Reproductive system and breast disorders	1	1	2	2	>0.9999
Respiratory, thoracic and mediastinal disorders	1	1	0	0	>0.9999
Rhinitis infective	7	6.8	10	9.8	0.6136
Rhinorrhea	3	2.9	3	2.9	>0.9999
Rotator cuff injury	0	0	1	1	>0.9999
Serum amylase increased	11	10.7	4	3.9	0.1049
Shingles	0	0	1	1	>0.9999
Sinus bradycardia	1	1	5	4.9	0.2118
Sinusitis	1	1	0	0	>0.9999
Skin and subcutaneous tissue disorders	4	3.9	8	7.8	0.3730
Skin infection	1	1	1	1	>0.9999
Somnolence	1	1	2	2	>0.9999
Sore throat	1	1	2	2	>0.9999
Surgical and medical procedures	0	0	5	4.9	0.0594
Syncope	4	3.9	2	2	0.6828
Testicular disorder	0	0	1	1	>0.9999
Thromboembolic event	0	0	2	2	0.4975
Tooth infection	1	1	2	2	>0.9999
Toothache	2	1.9	1	1	>0.9999
Tremor	2	1.9	0	0	0.4975
Upper respiratory infection	1	1	3	2.9	0.6213
Urinary frequency	2	1.9	0	0	0.4975
Urinary incontinence	1	1	1	1	>0.9999
Urinary retention	0	0	1	1	>0.9999
Urinary tract infection	11	10.7	11	10.8	>0.9999
Urinary urgency	3	2.9	1	1	0.6213
Urine discoloration	1	1	0	0	>0.9999
Urine output decreased	1	1	0	0	>0.9999
Urticaria	1	1	0	0	>0.9999
Vaginal infection	0	0	1	1	>0.9999

Vaginal inflammation	1	1	1	1	>0.9999
Vascular disorders	1	1	1	1	>0.9999
Vertigo	1	1	1	1	>0.9999
Vestibular disorder	0	0	1	1	>0.9999
Vision decreased	0	0	1	1	>0.9999
Vomiting	12	11.7	10	9.8	0.8220
Watering eyes	0	0	1	1	>0.9999
Weight loss	11	10.7	0	0	0.0007
Wound complication	1	1	0	0	>0.9999

Supplementary Table 6. Other secondary outcomes in ELAD.

**Variable**	**Visit**	**Placebo N=102**	**Treatment N=102**	**Between-group difference (95% CI)**	**p-value**
Composite Region Volume (mm^3)	Baseline	4556.03±787.64	4591.35±772.80		
	52 weeks	4389.58±746.94	4434.80±813.08	-3.97 (-76.00, 68.07)	0.91
Ventricular Volume (mm^3)	Baseline	58257.97±25282.35	53346.67 ±23247.87		
	52 weeks	61676.69±24914.04	56384.22 ±22923.22	-981.61 (-2170.40, 207.19)	0.10
Isthmus Cingulate (mm^3)	Baseline	2234.10±376.61	2203.32±357.10		
	52 weeks	2163.99±381.01	2171.12±338.55	39.61 (-5.61, 84.84)	0.09
Temporal Lobe (mm^3)	Baseline	48094.96±6226.95	48013.11±6575.59		
	52 weeks	46142.15±6270.73	46602.87±6868.07	696.24 (184.37, 1208.12)	<0.001
Parietal Lobe (mm^3)	Baseline	94545.44± 12930.04	93131.46±12788.30		
	52 weeks	91738.79± 13315.06	92184.39± 13745.87	1978.90 (360.12, 3597.67)	0.018
Frontopariet al (mm^3)	Baseline	241309.79± 28951.68	237843.15± 26524.65		
	52 weeks	235256.38±28958.10	236082.93± 27528.90	4271.526462 (722.41,7820.64)	0.02
Whole Grey Matter (mm^3)	Baseline	554657.85 ±55777.26	552493.01 ±53410.89		
	52 weeks	541102.11±58001.16	542666.83 ±55161.52	7274.42 (2704.05, 11844.8)	0.002

ANCOVA model adjusted for randomisation factors (age and MMSE as continuous variables) and baseline values of the outcomes.

A significance level of 0.05 was used to declare statistical significance for other secondary outcomes. A predefined significance level of 0.01 was used to declare statistical significance for any exploratory analyses. 95% confidence intervals (CI) are reported throughout.

Supplementary Table 7. Exploratory objectives in ELAD.

Variable	Visit	Placebo N=102	Treatment N=102	Between-group difference (95% CI)	p-value
Frontal Lobe VBM	Baseline	$33.13 \times 10^{-2} \pm 2.9 \times 10^{-2}$	$32.96 \times 10^{-2} \pm 3.44 \times 10^{-2}$		
	52 weeks	$32.38 \times 10^{-2} \pm 2.94 \times 10^{-2}$	$32.42 \times 10^{-2} \pm 3.43 \times 10^{-2}$	2.82×10^{-3} (2.10×10^{-4}, 5.44×10^{-3})	0.036
Hippocampus VBM	Baseline	$46.01 \times 10^{-2} \pm 6.78 \times 10^{-2}$	$46.43 \times 10^{-2} \pm 7.09 \times 10^{-2}$		
	52 weeks	$43.79 \times 10^{-2} \pm 6.42 \times 10^{-2}$	$44.73 \times 10^{-2} \pm 7.35 \times 10^{-2}$	5.54×10^{-3} (9.03×10^{-4}, 1.02×10^{-2})	0.021
Medial Temporal Lobe VBM	Baseline	$44.62 \times 10^{-2} \pm 5.68 \times 10^{-2}$	$45.07 \times 10^{-2} \pm 6.47 \times 10^{-2}$		
	52 weeks	$42.56 \times 10^{-2} \pm 5.40 \times 10^{-2}$	$43.51 \times 10^{-2} \pm 6.67 \times 10^{-2}$	5.16×10^{-3} (9.85×10^{-4}, 9.33×10^{-3})	0.017
Parietal Lobe VBM	Baseline	$32.24 \times 10^{-2} \pm 2.95 \times 10^{-2}$	$32.23 \times 10^{-2} \pm 3.50 \times 10^{-2}$		
	52 weeks	$31.47 \times 10^{-2} \pm 2.97 \times 10^{-2}$	$31.65 \times 10^{-2} \pm 3.63 \times 10^{-2}$	2.61×10^{-3} (5.9×10^{-5}, 5.16×10^{-3})	0.047
Temporal Lobe VBM	Baseline	$38.46 \times 10^{-2} \pm 3.67 \times 10^{-2}$	$38.54 \times 10^{-2} \pm 4.49 \times 10^{-2}$		
	52 weeks	$36.99 \times 10^{-2} \pm 3.65 \times 10^{-2}$	$37.41 \times 10^{-2} \pm 4.57 \times 10^{-2}$	3.90×10^{-3} (8.27×10^{-4}, 6.98×10^{-3})	0.014
Whole Grey Matter VBM	Baseline	$34.63 \times 10^{-2} \pm 2.95 \times 10^{-2}$	$34.55 \times 10^{-2} \pm 3.45 \times 10^{-2}$		
	52 weeks	$33.67 \times 10^{-2} \pm 2.94 \times 10^{-2}$	$33.84 \times 10^{-2} \pm 3.49 \times 10^{-2}$	2.98×10^{-3}	0.016

				($5.91x10^{-4}$, $5.36x10^{-3}$)	
Whole White Matter VBM	Baseline	$23.80x10^{-2}$± $3.77x10^{-2}$	$23.66x10^{-2}$± $4.27x10^{-2}$		
	52 weeks	$22.76x10^{-2}$± $3.71x10^{-2}$	$22.88x10^{-2}$± $4.33x10^{-2}$	$3.22x10^{-3}$ ($9.30x10^{-4}$, $5.51x10^{-3}$)	0.007

ANCOVA model adjusted for randomisation factors (age and MMSE as continuous variables) and baseline values of the outcomes.

A significance level of 0.05 was used to declare statistical significance for other secondary outcomes.

A predefined significance level of 0.01 was used to declare statistical significance for any exploratory analyses. 95% confidence intervals (CI) are reported throughout.
